# A Review of Green Synthesis of Metal Nanoparticles Using Algae

**DOI:** 10.3389/fmicb.2021.693899

**Published:** 2021-08-26

**Authors:** Abhishek Mukherjee, Dhruba Sarkar, Soumya Sasmal

**Affiliations:** ^1^Department of Biotechnology, Heritage Institute of Technology, Kolkata, India; ^2^Department of Biochemistry, School of Medicine, Vanderbilt University, Nashville, TN, United States; ^3^Department of Biological Sciences and Engineering, Netaji Subhas University of Technology, New Delhi, India

**Keywords:** nanoparticles, biosynthesis, algae, bio-nano factories, environment-friendly, psycho nanotechnology

## Abstract

The ability of algae to accumulate metals and reduce metal ions make them a superior contender for the biosynthesis of nanoparticles and hence they are called bio-nano factories as both the live and dead dried biomass are used for the synthesis of metallic nanoparticles. Microalgae, forming a substantial part of the planet’s biodiversity, are usually single-celled colony-forming or filamentous photosynthetic microorganisms, including several legal divisions like Chlorophyta, Charophyta, and Bacillariophyta. Whole cells of *Plectonema boryanum* (filamentous cyanobacteria) proved efficient in promoting the production of Au, Ag, and Pt nanoparticles. The cyanobacterial strains of Anabaena flos-aquae and *Calothrix pulvinate* were used to implement the biosynthesis of Au, Ag, and Pt nanoparticles. Once synthesized within the cells, the nanoparticles were released into the culture media where they formed stable colloids easing their recovery. *Lyngbya majuscule* and *Chlorella vulgaris* have been reported to be used as a cost-effective method for Ag nanoparticle synthesis. Dried edible algae (*Spirulina platensis*) was reported to be used for the extracellular synthesis of Au, Ag, and Au/Ag bimetallic nanoparticles. Synthesis of extracellular metal bio-nanoparticles using *Sargassum wightii* and *Kappaphycus alvarezi* has also been reported. Bioreduction of Au (III)-Au (0) using the biomass of brown alga, *Fucus vesiculosus*, and biosynthesis of Au nanoparticles using red algal (*Chondrus crispus*) and green algal (*Spyrogira insignis*) biomass have also been reported. Algae are relatively convenient to handle, less toxic, and less harmful to the environment; synthesis can be carried out at ambient temperature and pressure and in simple aqueous media at a normal pH value. Therefore, the study of algae-mediated biosynthesis of metallic nanoparticles can be taken toward a new branch, termed phyco-nanotechnology.

## Introduction

Green synthesis has become a reliable, sustainable, and ecological protocol for the synthesis of numerous nanomaterials such as metal oxides, hybrids, and bio-inspired materials in the field of materials science (Singh et al., 2018). Metallic nanoparticles are already intriguing scientists for more than a century and are now utilized widely in biomedical sciences and engineering (Lespes et al., 2020). Due to their anomalous size (length spanning within 1–200 nm) and shape-dependent properties and attractive applications in medicine, biofuel production, catalysis, electronics, and biotechnology, the synthesis of metal nanoparticles is a major area of research in nanotechnology (Ganesan et al., 2020). Green synthesis is thus considered to be an important tool to reduce the ill side effects of nanoparticles commonly used in laboratories and industry by conventional synthesis methods. The present review discussed all the established green synthesis methods used worldwide but focused on algae-based green synthesis only.

Among various microorganisms, microalgae are primitive microscopic plants, and they have significant advantages as cell factories for the production of nanoparticles compared to larger plants. Algae are aquatic filamentous photosynthetic organisms that fall under the kingdom of *plantae*. All these algae are broadly classified into two types: microalgae and macroalgae (Leaf et al., 2020). Macroalgaes can be counted under the naked eye whereas microscopes are required to observe microalgae. Unlike most biomass, both the algaes can be harvested several times in a single year. Algae also have the ability to grow without the help of any addition of outside chemicals or fertilizers. Microalgae grow extremely quickly and, on average, double their mass 10-fold faster than higher plants. It is known that various species of microalgae reduce metal ions. Algae are recognized as primitive microscopic plants and have few advantages, such as growth rate and nutrient requirements, for producing nanoparticles compared to higher plants (Jacob et al., 2021). The present review makes an attempt to improve researchers’ knowledge of green synthesis methodology of nanoparticle synthesis using different algal species and their various pros and cons.

## Classification of Nanomaterials and Methods of Synthesis

The worldwide biocompatibility of green nanoparticles and their enormous potential for use like catalysts, antimicrobials, biofuel generation, cancer/gene therapy, and sensorization have been attracting extensive interest. It is fascinated by the inherent proprietors of nano-materials like particular form, shape, composition, larger volume/superficial area, and the purity of the single constituents. Nanoparticles are characterized by their synthesis process. For the synthesis of nanoparticles, there are several methods available. Synthesis by using physical and chemical methods are quite costly and produce toxic by-products. For nanoparticle synthesis, two approaches exists: bottom-up and top-down (Shukla et al., 2021). The first step is the attrition of large macroscopic particles. The most common technique employing the role of the top-down approach for nanomaterial synthesis is the lithographic interferometric (Agarwal et al., 2017). This technique involves the synthesis by self-assembly of nanoparticles of miniaturized atomic components. It is a relatively inexpensive approach. It is based on the approach to the kinetic and thermodynamic balance. Among the various synthesis methods, the green synthesis methods are more promising as they will not produce any hazardous chemicals or leave almost negligible amount of waste products and utilizes environment-friendly chemicals for the synthesis of nanoparticles.

### Bio-Based Mechanism of Nanoparticle Synthesis

Biosynthetic methods are classified into intracellular and extracellular synthesis based on the location of nanoparticles produced (Öztürk, 2019). Extracellular production of nanoparticles, for example, is also being developed in order to further grasp the processes of synthesis, as well as to enable downstream processing and scale-up processing. Therefore, usage of algae for synthesis of inorganic nanoparticles has gained a lot of interest among researchers (Dahoumane et al., 2017). There are three major techniques used for synthesis of nanoparticles using algae. Apart from direct exploitation of live algae cells for nanoparticle synthesis, there are two other common methods: lysis of algal cells followed by extraction using different downstream process techniques such as centrifugation and filtration, and harvestion of nanoparticles from the supernatants of the algal broth (Dahoumane et al., 2017).

#### Algae-Assisted Synthesis

The field of processing algal biomass under catalytic conditions has received a lot of interest over the last decade. Algae should have a considerable economic significance in the future provided cost-effective upstream and downstream processing are developed. Algae are renowned for their capacity to hyper-accumulate heavy metal ions and remodel into more malleable shapes (Fawcett et al., 2017). Therefore, algae have been proposed as model organisms for fabricating bio-nanomaterials. The nucleation, development, and stabilization of nanoparticles are regulated by physical factors such as pH, precursor concentration, reaction time, exposure time, and temperature. These variables may be modified to adjust the size and morphology of the cells, as well as to avoid agglomeration. Carbohydrates, vitamins, nutrients, oil, fats, polyunsaturated fatty acids, bioactive compounds like antioxidants (polyphenols and tocopherols), pigments like carotenoids (carotene and xanthophyll), chlorophylls, and phycobilins are all found in algal extracts (phycocyanin and phycoerythrin) in different levels of concentration depending on alga species and its age. These active compounds have been described theoretically as nanoparticle synthesis reducing and stabilizing agents (Fawcett et al., 2017). The synthesis of nanoparticles from a wide diversity of algal resources proved to be one of the recent and most innovative areas of biochemical research as they have the property of reducing metal ions (Ponnuchamy and Jacob, 2016). Nanoparticles can be symthesized either intra- or extracellularly depending on the algal species and mode of operation. The different species of algae and the nanoparticles brought out are tabulated in Table 1. The table clearly depicts that algae (irrespective of species) can be used for the production of metallic nanoparticles.

**TABLE 1:** List of nanoparticles synthesized by different algae and their size and shape.

**Algae**	**Nanoparticles**	**Size**	**Shape**	**References**
*Bifurcaria bifurcate*	CuO	5–45 nm	Spherical and elongated	Abboud et al., 2014
*Galaxaura elongata*	Au	3.85–77.13 nm	Spherical	Abdel-Raouf et al., 2017
*Sargassum plagiophyllum*	AgCl	18–42 nm	Spherical	González-Ballesteros and Rodríguez-Argüelles, 2020
*Cyanobacterium Oscillatoria limnetica*	Ag	3.30–17.97 nm	Quasi spherical	Hamouda et al., 2019
*Caulerpa racemose*	Ag	5–25 nm	Spherical and triangle	Aboelfetoh et al., 2017
*Ulva fasciata*	ZnO	77.81	Spherical	Alsaggaf et al., 2021
*Turbinaria conoides*	Au	6–10 nm	Tri-spherical, triangle, and pseudo-spherical	Rajeshkumar et al., 2013
*Jania rubens*	Fe_3_O_4_	22.22–33.33 nm,	Spherical	Salem et al., 2020
*Portieria hornemannii*	Ag	70–75 nm	Spherical	Fatima et al., 2020

Algae, especially microalgae, are the new and up-and-coming organisms used for synthesis of nanomaterials. The choice of algae as a nanomaterial synthesizing agent is more promising than other living organisms or biomaterials. The researchers used different methods such as open cultivation systems (e.g., open ponds, tanks, and raceway ponds) and closed cultivation systems (e.g., photo bioreactors) to cultivate various algae species (Narala et al., 2016).

The following main steps are observed for the biosynthesis of metal nanoparticles using algae in the majority of the experiments:

(i) Heating or boiling an algal extract in water or an organic solution for a specified period of time.
(ii) Preparation of ionic metallic compound molar solutions.
(iii) Both algae and solutions of ionic metallic compounds are incubated for a definite period of time under controlled conditions, either with regular stirring or without stirring.

Based on characteristics of algae, nanoparticle synthesis can be accomplished by either an extracellular or an intracellular method in a precise and quantitative manner. The possible extracellular metallic nanoparticle production has been proposed to be attributed to polysaccharides, reducing carbohydrates, proteins, peptides, or other reducing factors present in the algal culture, and precipitating reducing metal ions to nanoparticles (Gahlawat and Choudhury, 2019). Both photosynthesis and respiration activity in algae are responsible for reduction of metallic ions, which further lead to formation of intracellular production of metallic nanoparticles. The enzyme nitrogenase has been proposed to have a function in the reduction of nanometals that occurs in cyanobacteria. The synthesis process was reported by participating in a photosynthetic electron transport system (PETS) and respiratory electron transport system (ETS) present on the cell membrane and in the cytoplasm of algae (Dahoumane et al., 2014). Reducing agents such as NADPH or NADPH-dependent reductase have also been proposed to be a key component for the reduction of metallic ions to nanoparticles in cyanobacteria through energy-generating reactions within the electron transport system and redox reactions occurring at the thylakoids, cell membranes, and in the cytoplasm (Oza et al., 2012). The pH of a material impacts on change of metallic nanoparticles concentration in solution. At a lower pH, the reduced power of the functional groups of the metallic nanoparticles is less relative to the pH greater than 6.5; however, the reduced power of the functional groups rises with higher pH values. After further increases in pH, the reduced power of the functional groups is not enough and hence cytotoxicity suffers. The hydrophobic and hydrophilic interactions between the nanoshells can inhibit the aggregation of the nanoparticles.

Cyanobacteria *Nostoc ellipsosporum* were first utilized in the laboratory for the intracellular biosynthesis of gold nanorods. After discovering healthy growers of 15 mg L^–1^ gold (III) solution (pH 4.5), the nanorods were formed inside cells at 20°C for 48 h (Parial and Pal, 2015). Gold nanoparticles (AuNPs) may be easily synthesized using a variety of processes for a variety of industrial and medical uses. However, due to their vast size distribution and strong aggregation property, their effectiveness as a catalyst has not been adequately researched and optimized.

The nutrient demands and maintenance cost of growth conditions such as light and temperature for the growth of algal cells are less than any prokaryotic and eukaryotic species. Many algae growing in a polluted environment with both metals and non-metals demonstrate that they are able to resist large quantities of metal or non-metal materials. In order to minimize and enhance their survival in higher concentrations of metals and the toxic effects of metal ions, algae have effectively developed defense-related mechanisms. Algae make excellent bio-sorbents due to their abundance in both seawater and fresh water, their cost-effectiveness, reusability, and their high metal sorption capabilities. The theoretical equilibrium model for bio-sorption (Langmuir model and Freundilich isotherm model) performs well in describing and forecasting metal uptake processes (He and Chen, 2014). The mechanisms of toxicity on algae (*P. subcapitata*) were also assessed and reported that higher sensitivity of the algal growth inhibition was observed by metalbased nanoparticles (Aruoja et al., 2015). The toxic effects of zinc oxide nanoparticles were studied on two marine algae *Tetraselmis suecica* and *Phaeodactylum tricornutum.* The study reported zero observable effect on growth inhibition by zinc oxide nanoparticles (Li et al., 2017).

Microalgal live cells are used to synthesize metallic nanoparticles in a one-step process that includes an aqueous solution containing metallic salts that is applied directly to the cells as they are being cultured. After being synthesized, the nanoparticles are released into the culture medium wrapped inside the matrix, which is often responsible for the formation of colloids. Due to its weight, the latter settles in the photo bioreactor. Repetitive cycles for nanoparticle biosynthesis may be conducted if needed by inserting fresh culture medium. Furthermore, microalgae preserved their nanoparticle biosynthetic capability when entrapped within organic vesicles. Several micro-algal organisms have been used in the biosynthesis of nanomaterials by extracting biomolecules from its cell.

Since silver nanoparticles are commonly used as antibacterial agents, a novel idea is needed for the effective application of silver nanoparticles as therapeutic agents to unknown diseases and infections. Silver nanoparticles have important advantages in biomedicine due to their physical and chemical flexibility. Although various chemical and biochemical methods still exist, an improved version of silver nanoparticles for extended applications in an environmentally friendly manner is urgently needed (Shanmuganathan et al., 2019).

Silver has been used for decades. Silver and silver nanoparticles are widely used in hard surface products and textiles, and are used in a broad variety of pharmaceutical, food industry, and domiciliary applications. Silver nanoparticles, which are well recognized for their antimicrobial function, may be synthesized by microalgae. Furthermore, the biomass generated in micro-algal culture for nanoparticle biosynthesis exhibits antimicrobial properties, as it can increase the antibacterial and antifungal ability of silver nanoparticles (Terra et al., 2019). Usage of silver nanoparticles was seen to extend to *C. vulgaris* conditioned media with a bright yellow to dark brown color shift (UV-Vis absorbance at 415 nm) (da Silva Ferreira et al., 2017).

### Application of Algae-Mediated Nanomaterials

Algae belonging to *Cyanophyceae, Chlorophyceae, Phaeophyceae*, and *Rhodophyceae* families have been used as nano-machines by intracellular and extracellular synthesis of gold (Au), silver (Ag), and other metallic nanoparticles. Algae are an attractive medium for the processing of diverse nanomaterials, owing to the inclusion of bioactive compounds in their cell extracts such as pigments and antioxidants that serve as biocompatible reductants. Silver nanoparticles synthesized in an environmentally friendly manner effectively inhibit bacterial growth by eliciting bactericidal activity against Gram-negative and Gram-positive biofilm-forming pathogens. As a result, silver nanoparticles produced by *G. amansii* (brown algae) may serve as potential anti-fouling coatings for a variety of biomedical and environmental applications (Pugazhendhi et al., 2018). Nanoparticles produced by algae can compete with standard drugs and have been shown to have antibacterial, anticancer, and antifungal effects. Aside from medical uses, metal nanoparticles have a broad variety of applications in computing, optics, cosmetics, and other areas.

#### Antioxidant Activity

Defatted algal biomass was reported for synthesis of silver nanomaterial by Chokshi et al. (2016). After lipid extraction, the residual biomass of the microalgae *Acutodesmus dimorphus* was used by the researcher to make a micro-algal water extract, which was then used to make silver nanoparticles (with a scale of 2–20 nm). The antioxidant ability of the biosynthesized silver nanoparticles was assessed using 2,2′-azino-bisphosphate (3-ethylbenzothiazoline-6-sulfonic acid) (Chokshi et al., 2016).

#### Antibacterial Activity

Antibacterial behavior of nanoparticles synthesized from algae has been studied against a variety of bacterial strains. Silver nanomaterials manufactured from the brown seaweed *Padina tetrastromatica* effectively slowed the growth of *P. aeuroginosa, Klebsiella planticola*, and *Bacillus subtilis* (Sangeetha et al., 2012). Another research found that robust and colloidal-shaped silver nanomaterials made from an aqueous extract of the green marine algae *Caulerpa serrulata* had excellent antimicrobial activity against *Shigella* sp., *S. aureus*, *E. coli*, *P. aeruginosa*, and *Salmonella typhi* at lower concentrations. *E. coli* had the largest zone of inhibition of 21 mm, while *S. typhi* had the smallest zone of inhibition of 10 mm at 50 μl solution of silver nanomaterials (Aboelfetoh et al., 2017).

#### Antifungal Activity

The antifungal function of nanoparticles is defined by their size and form. The broad surface region of small nanoparticles ensures that microbial growth is inhibited. The enhanced contact area of the spherical form with size-reduced ions eliminates growth of fungus.

## General Characterization Techniques

A range of analytical techniques such as transmission or scanning electron microscopy (TEM or SEM), atomic force microscopy (AFM), dynamic light scattering (DLS), x-ray photoelectron spectroscopy (XPS), powder x-ray diffractometric (XRD), Fourier infrared transform spectroscopy (FTIR), and UV-Vis spectroscopy are used to characterize metal and non-metal nanoparticles. The aforementioned techniques are used to characterize the morphology of the nanoparticles, including particle size, shape, crystallinity, fractal dimensions, pore size, and surface area. The image of nanoparticles using different image capturing instruments and their versatile applications are tabulated in Table 2.

**TABLE 2:** Different imaging techniques used in nanoparticles.

**Imaging type**	**Type of nanoparticle used**	**Applications**	**References**
Magnetic resonance imaging (MRI)	Gd_2_Hf_2_O_7_ nanoparticles	Chemo-/photo-thermal-/radiotherapy of resistant tumors	Kuang et al., 2020
Fluorescence-based imaging (FBI)	Carbon quantum dots with gold nanoparticles	Detection of aldicarb	Sajwan et al., 2021
Confocal microscopy	Zinc oxide nanoparticle	Check sunscreen toxicity	Yamada et al., 2020
Transmission electron microscopy (TEM)	V_2_O_5_ nanoparticles	Photocatalytic and antibacterial studies	Karthik et al., 2020
Scanning electron microscopy (SEM)	Silver nanoparticles	To check *in vitro* anti-acne activity	Srivastava et al., 2020
Reflection electron microscopy (REM)	Germanium nano islands	Nucleation control	Shibata et al., 2000

The change in color of a mixture formed at a particularly specific temperature has been utilized as a visual indication for the synthesis of silver and gold non-metals in certain instances. A shift of the color of the reaction mixture to brownish violet or a change to violet suggests the formation of silver nanoparticles, and a change of the reaction mixture to purple or pink indicates the formation of gold nanoparticles (AuNPs). The biosynthesis of nanometals in the aqueous process is monitored using absorption spectroscopy in the UV-Vis spectral range (190–1,100 nm) since nanometals have striking optical properties due to surface plasmon resonance (SPR) (Borah et al., 2020). The combined motions of the free electrons of metal nanoparticles in resonance with the light wave produce a special SPR absorption band (λ_*max*_) that is dependent on size, shape, aspect ratio, and the dielectric constant of the metals. As the particle size of the nanomaterial grew in the aqueous solution, the bandwidth dropped with the increased band amplitude; hence, a UV-Vis spectrophotometer may be used to determine the particle size of the metal nanoparticles in the aqueous solution. Blue and green light rays, which have a lower intensity and are more diffuse, are employed in a broad spectrum band (between 320 and 580 nm) (Gopu et al., 2021).

Biomolecules present in algal extracts, such as polysaccharides, peptides, and pigments, can play an important role in the formation of biomolecular complexes for bio-mining the metals. These materials are often observed to be responsible for stabilizing and capping metal nanoparticles that are created by the use of amino-derived or cysteine-conjugated polysaccharides, while polysaccharides are used for metal core stabilization and capping. The reducing agents responsible for the reduction, stabilization, and capping of metal nanoparticles may be identified using Fourier transform infrared (FTIR) spectrometry. Through using the FTIR spectra, it is understood that functional groups such as the -C = O-, -NH_2_, and -SH- groups conform to the surface of the biosynthesis non-metals (Huq, 2020).

To illustrate the development of various sizes of nanoparticles, TEM and SEM are majorly used by researchers. SEM imaging needs a prolonged preparation phase, which is often completed by metal coating to minimize charging artifacts and rapid radiation damage to biomaterials during the imaging process. Besides this, electron diffraction (EDX) machine is also paired with SEM and TEM for better identification. Samples are prepared by drop coating the metal nanoparticle solution onto carbon copper grids followed by drying the grids in preparation for measurement. X-ray diffraction (XRD) study will reveal the crystalline structure of nano-metals. Energy-dispersive spectroscopy (EDS) is also a very common instrument to determine the presence of metal (Abbasi et al., 2020). Typically, EDS systems are integrated into either a SEM or an EPMA equipment. A sensitive x-ray detector, a liquid nitrogen dewar for cooling, and software for collecting and analyzing energy spectra are all included in an EDS system. Through these methods, researchers can easily quantify the structural characteristics of nanomaterials.

## Conclusion

Algae, like other biological species such as mushrooms, yeast, and bacteria, have significant nanoparticle synthesis effects. Algal-based nanosynthesis has developed into a separate branch known as phyco-nanotechnology. Various studies on the biosynthesis of nanoparticles using seaweed extracts have been conducted. However, the use of microalgae for nanoparticle synthesis is very limited. In this regard, several recent experiments have shown that microalgae can be used to synthesize metal nanoparticles. Both micro- and macroalgae are the frontrunners in the development of nanoparticles that can effectively provide a range of applications. Algae are also common in agriculture. When seaweeds (macroalgae) are used as fertilizers, there is less nitrogen and phosphorus runoff than when animal manure is used. Algae are recognized for their capacity to hyperaccumulate heavy metal ions and remodel into more malleable shapes. Because of these appealing characteristics, algae have been proposed as model organisms for the processing of bio-nanomaterials.

Different forms of algae that have not been extensively researched will be used to study the processes of nanoparticle synthesis. To identify the proteins and enzymes involved in algal nanoparticle synthesis, extensive research would be needed to understand the exact mechanisms of the reaction. Designing easy and low-cost techniques will make the synthesis process commercially viable. Nano-biotechnology has the ability to revolutionize human health, as well as the agriculture and food markets, by offering new methods for disease prevention and identification. As a result, useful methods for investigating the biological capacity of algae and blue-green algae for nanoparticle synthesis should be established, as well as their behavior and aggregation in animals and plants.

Future studies should look into the size distribution and chemical structure of nanoparticles made from algae. Overall, nano-biotechnology that uses algae and blue-green algae to synthesize nanomaterials is still in its early stages, and further research and development is required.

## Author Contributions

All authors wrote, reviewed, and approved this manuscript for publication.

## Conflict of Interest

The authors declare that the research was conducted in the absence of any commercial or financial relationships that could be construed as a potential conflict of interest.

## Publisher’s Note

All claims expressed in this article are solely those of the authors and do not necessarily represent those of their affiliated organizations, or those of the publisher, the editors and the reviewers. Any product that may be evaluated in this article, or claim that may be made by its manufacturer, is not guaranteed or endorsed by the publisher.
